# Landscape of Cellular Bioeffects Triggered by Ultrasound-Induced Sonoporation

**DOI:** 10.3390/ijms231911222

**Published:** 2022-09-23

**Authors:** Dawid Przystupski, Marek Ussowicz

**Affiliations:** Department of Paediatric Bone Marrow Transplantation, Oncology and Haematology, Wroclaw Medical University, 50-556 Wroclaw, Poland

**Keywords:** sonoporation, ultrasound, cancer cells, membrane permeabilization, membrane resealing, calcium signalling, cell death, oxidative stress, mechanotransduction, DNA damage

## Abstract

Sonoporation is the process of transient pore formation in the cell membrane triggered by ultrasound (US). Numerous studies have provided us with firm evidence that sonoporation may assist cancer treatment through effective drug and gene delivery. However, there is a massive gap in the body of literature on the issue of understanding the complexity of biophysical and biochemical sonoporation-induced cellular effects. This study provides a detailed explanation of the US-triggered bioeffects, in particular, cell compartments and the internal environment of the cell, as well as the further consequences on cell reproduction and growth. Moreover, a detailed biophysical insight into US-provoked pore formation is presented. This study is expected to review the knowledge of cellular effects initiated by US-induced sonoporation and summarize the attempts at clinical implementation.

## 1. Introduction

According to the World Health Organization, cancer is the second-leading cause of death in the world. In 2015, approximately 8.8 million people died from neoplastic diseases in the United States [1]. Despite advances in cancer research and clinical studies, many patients succumb to disseminated, recurrent or therapy-resistant disease [2]. Although many improvements have been made in oncological treatment over the last years, cancer-related deaths occur in 30–50% of all cancer patients [3]. Mortality rates remain very high, especially for solid tumors such as pancreatic, colon, cervix, and brain cancers [4]. One of the obstacles in the efficient therapy is insufficient intracellular concentration of anticancer medications due to various mechanisms of drug resistance developed by cancer cells [5]. Moreover, standard chemotherapy usually damages not only tumor cells, but also healthy tissues and leads to serious adverse effects such as immunosuppression, cardiotoxicity, or kidney failure [6,7], which strongly limit clinically available doses of approved cytostatics for cancer treatment. Enhancing on-target drug activity and reducing off-target effects are the goals of novel anti-cancer therapies [8]. For such reasons, in recent years, much attention has been paid to research concerning drug transport into cells. Alternative methods of controlling drug release and deposition such as light, magnetic and electric fields, as well as neutron beams, have been gaining increasing attention [9]. Interestingly, sound waves can also be used for targeted drug administration.

**Figure 1:**
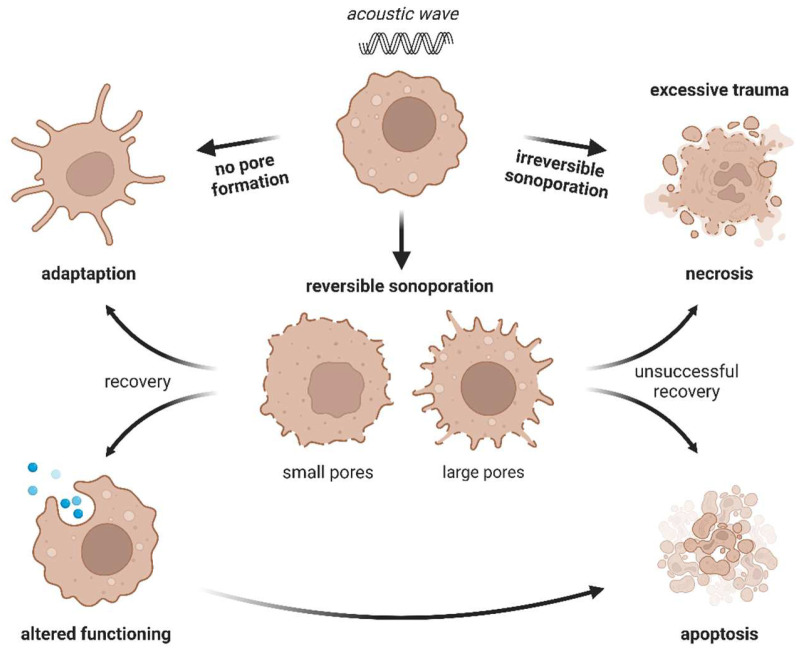
Schematic representation of possible cellular outcomes induced by sonoporation. As the acoustic wave approaches the cell, small or large pores in the cell membrane are generated. When membrane resealing is not possible and cell injury is too hard (irreversible sonoporation), then necrosis occurs. On the other hand, if the cell can recover after ultrasound (US) exposure, this process may alter cell functioning and when adaptation is not possible then apoptosis will be initiated. Created with BioRender.com.

Ultrasound (US) has been widely implemented in medicine over the last half century, mainly for diagnostic purposes, beginning with the monitoring of pregnancy and ending with a detailed examination of soft tissues and blood vessels. Since US can be focused, acoustic waves are easily able to deliver kinetic energy to selected small volumes deep within a human body without damaging other tissues. Therefore, physicians or physical therapists used them to stimulate healing and soften scar tissue, or high-intensity focused US—HIFU—to damage prostate cancer or break down kidney stones [10,11,12,13]. However, a completely new chapter in US research was opened in 1976 when a strong cytostatic effect of nitrogen mustard was observed in L1210 leukaemia cells treated with sonication without mechanical damage to cells [14]. Later discoveries and advances in the field of US have led to the idea of using this technology as a support for chemotherapy. This was supposed to provide greater drug distribution within tissues. In that way, scientists began exploring US as a new tool for delivering various substances to cells. Until now, many studies have shown that US may improve drug uptake by causing small pores in the cell membrane. US-triggered cell membrane permeabilization, also called sonoporation, is a process that can be reversible or irreversible (Figure 1). Although reversible sonoporation (RS) improves cellular drug concentration without causing direct cell death, irreversible sonoporation (IRS) is believed to be lethal for cells and cause in most cases their immediate necrosis [12,15,16,17,18]. Furthermore, these phenomena are considerably intensified in the presence of gas-filled microbubbles. When acoustic waves approach microbubbles, the mechanical interactions between US and microbubbles generate much stronger forces, which maximize US-triggered bioeffects [19,20,21].

Since sonoporation disrupts cell membrane homeostasis by causing physical wounding of its structure, it is likely that US also alters the functioning of sonoporated cells, even if permeabilization is temporary. Taking into account the fact that the mechanical effects induced by US are originally observed on the cell membrane as membrane distortion, the extracellular kinetic energy released by US can be transduced deep into the cell by complex mechanotransduction pathways and thus modify the function of the cell [22]. Until now, numerous studies have reported various cellular alterations caused by US, e.g., a decreased viability [23], disrupted cell membrane potential [24,25], altered calcium signalling [26,27], production of reactive oxygen species [28,29] or generation of shear stress [30,31]. These and other US-triggered mechanisms are meaningful bioeffects, which strongly affect the intracellular environment disrupted by drug-mediated sonoporation and determine the final therapeutic outcome. Based on this research progress, the aim of this review was to assess and summarize the intracellular bioeffects caused by sonoporation and the related biological mechanisms involved in US-triggered permeability. Additionally, the general characteristic of the sonoporation phenomenon is firstly discussed, including information about the mechanisms of pore formation and the physical and biophysical parameters that determine the sonoporation efficacy. Finally, this article provides a commentary on the performed research and highlights the unexplored gaps that should be addressed in future studies.

## 2. Mechanisms Responsible for Sonoporation

US is an acoustic wave that creates a series of pressure fluctuations, which can even establish a spatial standing wave pattern when the reflecting constraints are used. Generally speaking, any biological effects that arise will have a direct correlation with the applied US parameters, the distance between exposed cells and the energy source, the transducer or any structures that generate a standing wave, as well as the presence of acoustically active microbubbles [32,33]. The occurrence of physical pores in the cell membrane after US exposure has been confirmed in several studies using scanning electron microscopy and atomic force microscopy [34,35,36,37]. Previous studies have reported that sonoporation can occur in the presence of microbubbles, which is called bubble-based sonoporation (BBS), or without them—non-bubble-based sonoporation (NBBS). Although both the mentioned mechanisms result in membrane permeabilization, the biophysical processes responsible for pore formation are noticeably different.

When acoustic waves spread in fluid-containing cells, these cells are subjected to the acoustic streaming-induced shear force and the acoustic radiation force at the same time. This environment can alter the integrity of the membrane and therefore generate pores in the cell membrane [38,39,40,41]. These phenomena were implemented to design various mechanisms that allow NBBS, such as ejecting cells through the nozzle orifices using traveling acoustic waves [42], exposing cells to standing acoustic waves in quarter-wavelength resonators [43,44] or applying Lamb waves to cells cultured on thin substrates [45,46,47]. Some researchers also used bulk acoustic waves, which were travelling above the transducer, to induce NBBS. Other possibilities open when the target cells are close to the source of US. In such a case, bulk acoustic waves can be focused in a small area to generate concentrated acoustic energy [48,49,50,51], or hyperfrequency bulk acoustic waves can be used to induce acoustic streaming and create pores in the cell membrane [52,53].

**Figure 2:**
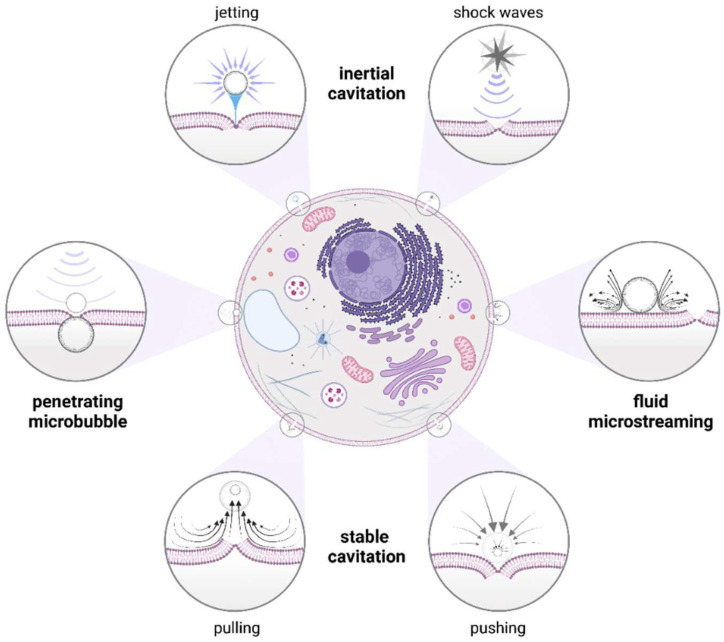
Schematic representation of physical mechanisms involved in sonoporation. Created with BioRender.com.

US contrast agents are typically microbubbles (MBs) that contain a gas-filled core encapsulated by a stabilization coating composed of lipids, polymers, or albumin. Due to an average size between 1 and 8 μm, they can easily pass through the capillaries around the human body [54,55,56]. By using MBs as cavitation nuclei, the cavitation threshold is reduced by enhanced absorption of acoustic energy that intensifies US-induced effects [57,58,59]. When exposed to US, microbubble responds in a wide range of behaviours that cause acoustic cavitation [60,61]. As its volume changes rapidly, acoustic cavitation results in a number of mechanical, chemical, and thermal phenomena, which dramatically change the local environment and lead to bubble-based sonoporation [62]. It is fundamental to note that different types of acoustic cavitation can occur depending on the intensity of the applied US known as the mechanical index. The mechanical index (MI) is defined as the peak negative pressure amplitude (MPa) divided by the square root of the centre frequency (MHz) of the acoustic wave [63]. The observed cellular bioeffects triggered by US are strongly dependent on MI. At low mechanical index (MI < 0.2), volume of MBs fluctuates continuously and generates pulling and pushing movements of the cell membrane that cause membrane ruptures [64]. This process is also called stable cavitation [65]. It is worth mentioning that when microbubbles are attached to the cell membrane, fluid movements around the fluctuating bubble can induce a local shear stress intense enough to rupture the cell membrane. This phenomenon is known as microstreaming [66]. In this situation, microbubble may also penetrate lipid bilayer causing membrane permeabilization. When MI is higher (MI > 0.2), the acoustic pressure is more extreme, so the released kinetic energy is much more powerful. In such a case, the MBs oscillate vigorously, leading to what is called inertial cavitation caused by their collapse and destruction, which generate shock waves or microjets penetrating the lipid bilayer [65,67,68]. The mechanical phenomena generated by microbubbles are shown in Figure 2. As US causes repeating compression (at higher pressure) and expansion (at lower pressure) movements of the molecules, the transmitted energy can significantly increase a medium temperature, which is responsible for the thermal effects of BBS. The rise in temperature is strongly correlated with the initial energy converted into the acoustic wave, leading to local hyperthermia [62]. This phenomenon is used for high-intensity focused US (HIFU)-ablation therapy of cancer [69]. Moreover, US-induced hyperthermia was claimed to increase drug uptake by modulating membrane permeability, especially in multidrug resistant cells [70]. Given that BBS is accompanied by severe mechanical and thermal effects, both may lead to the chemical phenomena happening at BBS. When the gas pressure increases significantly or the temperature rapidly rises during the collapse of MBs, such extreme conditions can generate reactive oxygen species (ROS) as well as electromagnetic waves, also known as sonoluminescence. Interestingly, both ROS and sonoluminescence are known to alter drug resistance [62,71,72,73].

## 3. Mechanical and Biological Factors Affecting US Efficacy

Numerous studies have reported that the intensity of permeabilization depends on a wide range of biomechanical factors. There is a considerable amount of literature showing the positive correlation between the characteristics of acoustic waves and the transfer efficiency. This effect was described for parameters such as acoustic pressure [13,74], acoustic energy [75,76], exposure time [76,77,78], pulse duration [20,76], MI [79] as well as duty cycle [76,80]. However, the published articles sometimes seem to contradict each other. For example, Karshafian et al. [76] showed that the increasing mechanical energy led to intensified cell death, especially for high-frequency acoustic waves such as 5 MHz. Unlike other research carried out in this area, Forbes et al. [81,82] revealed that exposures to 0.92, 3.2, and 5.6 MHz had almost the same impact on cell viability. Thus, the cellular response to US seems to be unique for a particular cell type, and detailed preliminary research regarding the range of US parameters should be performed.

In terms of acoustic wave effects being deeply investigated, much work concerning the effect of microbubbles has been carried out as well. Many commercial and self-made MBs were used in US-mediated experiments, providing various sonoporation severity depending on the size of the MB, the chemical composition, or the type of gas filling the core. Several studies have shown that the higher the concentration of MBs, the more intensified the sonoporation and the bigger the pores in the cell membrane [37,83]. Moreover, the reduced bubble-cell distance provided a higher transfer effectiveness. Le Gac et al. [84] noted that when the distance between the cell and the bubble was less than 75% of the microbubble radius, the probability of cell membrane perforation exceeded up to 75%. Another interesting factor that affects the intensity of the BBS is the viscosity of the fluid. Rosenthal et al. [72] mentioned that the radial motion of MB and the ability to damage cells by MB decreased when the viscosity of the fluid increased. Despite this, the effect of fluid viscosity on cell-based sonoporation has been poorly investigated and omitted in most research. Some papers also pointed out the crucial role of fluid composition. One of the most important components in extracellular fluid affecting sonoporation are calcium ions, which have been shown to be essential for membrane resealing after US exposure. This phenomenon is described in more detail in Section 4.3.

Although mechanical agents are commonly reported and well documented in many papers, the effect of biological factors on the intensity of sonoporation has been poorly discussed. Since cell structures and geometric and mechanical characteristics such as cell size, shape, or elasticity vary between different cell lines, it is no surprise and not unexpected that the intensity of permeabilization is also altered by cellular processes [59]. Few researchers have addressed the issue of sonoporation sensitivity in various cell lines. For example, Shi et al. [85] proved that the sonoporation conditions that provide effective sonoporation were different for four cancer cell lines derived from various tissues. Khayamian et al. [86] argued that US caused much more intense disruption of the cell membrane in healthy HUVEC cells than in MCF-7 cancer cells due to the particular cytoskeleton pattern of actin stress fibers. Furthermore, Jia et al. compared the effect of US on MCF-7 cells and doxorubicin resistant MCF-7/ADR cells. Interestingly, they found that MCF-7/ADR cells displayed a higher sensitivity to sonoporation [87]. Furthermore, transfection efficacy has also been shown to vary between different cell types [59]. These findings confirm that the final outcome of sonoporation strongly depends on the type of cell, the source of tissue, or the resistance status.

A few papers addressed the sonoporation intensity in cells that undergo a particular cell cycle phase. Pichardo et al. [88] showed that the cells at G2 and M phase had the highest transfection efficacy. One of the most likely explanations for this effect is the dependence of sonoporation efficacy on the biophysical characteristics of the cell. Some articles suggested that cell cycle progression is accompanied by cytoskeleton reorganization that significantly changes cell mechanical properties [89,90]. To confirm this, Fan et al. evaluated single-cell responses to sonoporation of HeLa cells synchronized in a particular cycle phase. On the basis of the AFM analysis, they noticed that cells in the S-phase having the smallest elastic modulus were the most susceptible to microbubble-mediated sonoporation. They suggested that intense microstreaming-induced shear stress was present in cells with a small elastic modulus that led to the highest sonoporation efficacy [91]. The chemical composition of the cell membrane also seems to be crucial for the sonoporation outcome. Jurak et al. [92] noticed that microbubbles are likely to interact with appropriate acoustically active areas of the cell membrane which are highly composed of lipid domains enriched with sphingomyelin and cholesterol that rigidize the cell membrane.

This is worth mentioning, as one of the biggest limitations of the US-related in vitro studies is the use of cell suspensions. It provides an easy way to obtain repetitive results, but the collected data may be clinically irrelevant because bubble activity or acoustic features differ considerably in cell suspensions and solid tissues. Furthermore, sonoporation is limited in tissues due to the lack of cavitation nuclei around most cells, but can be overcome by using microbubbles or other nanocarriers [93].

## 4. Cell Membrane

### 4.1. Membrane Permeabilization

As explained above, exposure to US results in the generation of pores in the cell membrane. Many researchers tried to determine the pore size hypothesizing that the pores are round-shaped formations with a particular diameter and on the assumption that passive diffusion is the most crucial mechanism for US-triggered transport of molecules into the cell. SEM studies provided direct evidence of membrane permeabilization that showed the presence of irregular pores with a diameter of 100 nm up to several micrometers [18,35,94,95]. Zarnitsyn et al. performed a comprehensive analysis of transmembrane transport through the cell membrane after sonoporation using DU145 prostate cancer cells. Their experimental studies confirmed the intracellular uptake of molecules up to 28 nm radius through membrane wounds where most uptake took place following sonication predominantly by diffusion and where the most effective loading was observed for smaller molecules. Furthermore, mathematical modelling predicted that membrane disturbances had a radius of 300 nm at the beginning and closed with half-lives of 20–50 s [96]. Zhou et al. monitored the sonoporation of single *Xenopus laevis* oocytes in real time by changes in the transmembrane current (TMC) under voltage clamp. In that way, they estimated that the mean pore radius was 110 nm [97]. Similar measurements were made for HEK-293 cells in the presence of microbubbles—in this case the maximum diameter of the pores was 100 nm, showing that MBS was able to generate larger pores [98]. Some other papers reported that the radius of the pores was 50–75 nm [35], 500 nm [21] and 500–2500 nm [37]. Interestingly, Duvshani-Eshet et al. [34] using AFM observed significant changes in cell surface topography and increased surface roughness after microbubble-based sonoporation. Additionally, SEM and AFM studies highlighted that longer time of US exposure and higher acoustic wave pressure created larger pores [37,99]. However, care must be taken with the interpretation of the images performed applying such methods. They mostly require preserved cells specially processed for imaging many hours after the experiment when the pore structure can be strongly disturbed at the time of analysis.

Some researchers also described the relationship between bubble diameter and bubble-to-cell distance with the degree of US-triggered permeabilization. For example, Qin et al. investigated the real-time single-cell response to sonoporation triggered by acoustic droplet vaporization (ADV). They indicated that large ADV bubbles induced irreversible sonoporation only if they were closely located to the cell, whereas the same bubbles far from the cell membrane were not able to induce sonoporation. Thus, this observation proved that the final outcome of sonoporation depended on the size of the microbubble and its distance to the cell; irreversible sonoporation was more likely when the bubble to the cell distance decreased as well as when the diameter of the bubble increased [16]. Furthermore, Hu et al. [100] discovered that the size of the generated pore determined the reversibility of the sonoporation: membrane perforations smaller than 30 μm^2^ disappeared within 1 min after US treatment, whereas pores >100 μm^2^ were still open within half an hour. Furthermore, the time of membrane resealing determined post-sonoporation cell viability: only cells with pores that resealed in a minute remained viable after US treatment [100,101,102]. It is worth mentioning that the size of the membrane wound during BBS depends on the sonoporation mechanism—small pores (up to a few hundred nanometers) are generated by inertial cavitation caused by microbubbles [35] whereas large pores (hundreds of nanometers up to several micrometres) result from stable cavitation activity of microbubbles [100,103,104].

Since US-triggered cell membrane permeabilization can be disturbed by multiple factors acting simultaneously such as the size of bubble, and its distance to cell or acoustic wave energy, the final sonoporation efficacy may differ within the population of the sonoporated cells at the same time. Some papers provided proves for this heterogeneity in particle uptake. Guzmán et al. [105,106] believed that this phenomenon was caused by unequal exposure of various cells to US and cavitation; however, they did not observe a whole distribution of uptake intensities within sonoporated cells. Moreover, De Cock et al. noticed only two populations of cells with different levels of molecule uptake. They analysed separated subpopulations of sonoporated cells using confocal microscopy and discovered that the cells displayed different uptake mechanisms: endocytic uptake for low uptake intensity and membrane permeabilization for high uptake intensity. Additionally, they mentioned that the higher the acoustic pressure, the more sonoporated cells with high uptake were generated. Thus, they concluded that endocytosis is promoted at low pressures, whereas pore formation predominates at high pressures, and that the uptake mechanism can be controlled by adjusting the acoustic wave pressure [107].

### 4.2. Cell Membrane Potential

The bioelectrical gradient provided by a well-organized balance between intra- and extracellular ions is responsible for the so-called cell membrane potential that affects the transmembrane transport of various molecules. When its baseline level is disturbed by particular stimuli, cell homeostasis and cell behaviour are affected. Since acoustic waves are capable of penetrating the cell membrane, they are also likely to alter the cell membrane potential [108]. Preliminary studies on this topic confirmed that TUS can disturb the membrane electric charge [20,24], especially in the presence of microbubbles [25,109,110]. Qin et al. investigated the plasma membrane potential of HeLa cervical cancer cells subjected to US. They noticed that irreversibly sonoporated cells displayed permanent depolarization of the cell membrane, but when the sonoporation was reversible the depolarization was transient or sustained. Moreover, they found that intracellular calcium ion concentration increased simultaneously during US exposure and returned to the initial level in the app. 100 s [108]. In contrast, Tran et al. [24] observed sonoporation-mediated hyperpolarization of MDA-MB-231 breast cancer cells that lasted as long as BBS was happening. As Qin et al. noted calcium influx during sonoporation. Going further, they proved that the observed cell membrane hyperpolarization was generated by sonoporation-triggered calcium ions influx, which opened calcium-gated BK_Ca_ stretch channels boosting potassium ion efflux [24]. These contradictory observations may be explained by the fact that HeLa cells are known for the lack of voltage-gated ion channels, which makes sonoporation-mediated hyperpolarization impossible. Thus, the composition of membrane ion channels is crucial for determining the effect of US on the potential of the cell membrane. It is also worth mentioning that other researchers noticed a change in transmembrane current only when BBS was performed [111].

### 4.3. Membrane Resealing

**Figure 3:**
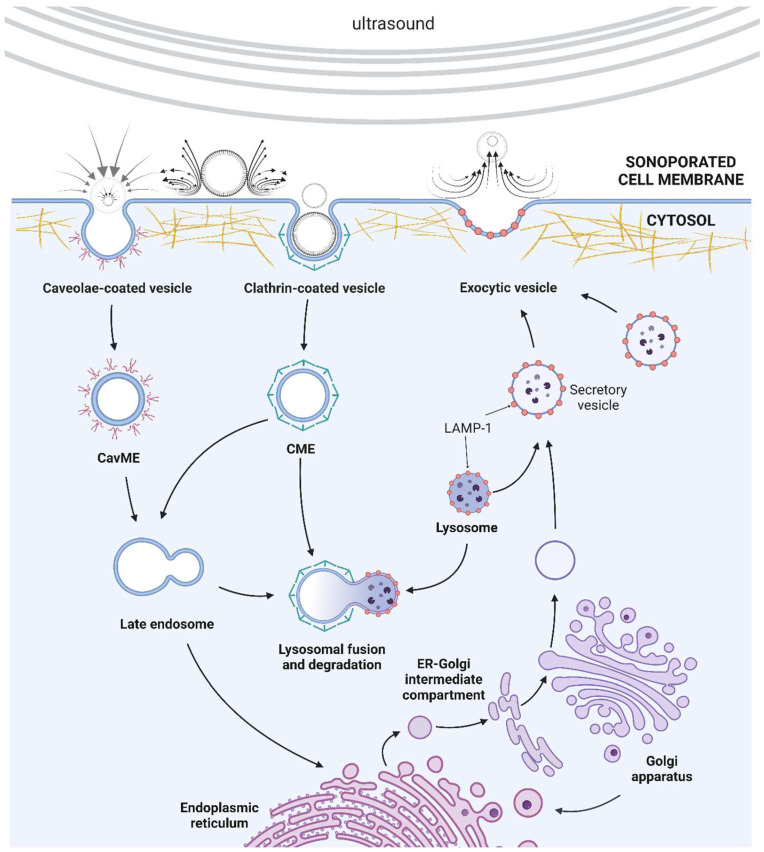
Membrane-resealing mechanisms triggered by US. Created with BioRender.com.

US-generated pores in the cell membrane must be repaired to ensure cell survival and limit the transmembrane passage of various extracellular or intracellular agents before pore resealing. Membrane repair is crucial to prevent intracellular accumulation of ions capable of disrupting cell homeostasis. According to previous studies, sonoporation is likely to induce two mechanisms that allow membrane resealing (Figure 3): endocytosis-triggered membrane repair and exocytosis-linked vesicular patching [12,112]. The mechanical forces released by US can induce cell-membrane deformation by compressing microbubbles onto the cell membrane, or stable cavitation creating microstreaming in the surrounding fluid. Such phenomena provide changes in cell-membrane tension without disrupting plasma-membrane integrity as well as induction of shear stress which altogether leads to a rearrangement of cytoskeletal fibres. These mechanical forces can be perceived by mechanosensors, such as integrins or stretch-activated ion channels that are capable of transducing such stimuli and changing cellular processes [113,114]. The processes of rebuilding (exocytosis) or removal (endocytosis) of the cell membrane have been claimed to restore the initial tension and integrity of the plasma membrane [12]. Endocytosis is believed to be mainly responsible for the resealing of small pores caused by oscillating microbubbles [12,115]. On the other hand, exocytosis and lysosomal patches repair large membrane pores generated by collapsing microbubbles [99,116]. Furthermore, only pores smaller than 0.2 µm can be successfully resealable [117]. This explains the contradictory results of different groups reporting only the occurrence of US-induced exocytosis or endocytosis in drug-uptake experiments.

Previous studies have shown that caveolae-mediated endocytosis (CavME) and clathrin-mediated endocytosis (CME) are triggered by US. CavME and CME occur through caveolae and clathrin-covered pits, then the created vehicles can fuse and form an early or late endosome, which could be united with lysosomes for degradation [115]. It is still unclear which endocytic pathway is predominant; however, Lionetti et al. [118] noticed that caveolae-mediated endocytosis provided much more intense uptake of fluorescent probe in HUVEC cells subjected to diagnostic-level US. To check whether US-mediated uptake was caused by sonoporation alone or endocytosis, the effect of inhibition of various endocytic pathways was studied. Endocytosis inhibitors have been found to significantly reduce molecule uptake and the clathrin-mediated pathway played an major role in uptake [79]. Meijering et al. [119] showed that large molecules such as 500 kDa dextran used in their studies were absorbed mainly by endocytosis, whereas the smaller dextrans (4 kDa) were absorbed by sonoporation as well as endocytosis. It is worth mentioning that sonoporation triggered endocytosis not only during US exposure but also many minutes after that. Derieppe et al. investigated US and SonoVue microbubble-mediated uptake of SYTOX Green, a 600 Da hydrophilic model probe in C6 rat glioma cells. They noticed that cavitation-induced endocytosis provided a continuous increase in intracellular SYTOX Green accumulation for 4 h that lasted for several hours [120]. Moreover, Tardoski et al. [121] showed the accumulation of the endocytic marker—clathrin—in the cytoplasm after US treatment and its concentration substantially reduced in 18 h. These findings proved that sonoporation can induce long-term and post-exposure molecule internalization that is responsible for the appearance of a substantial increase in particle uptake. Finally, antioxidants have been reported to decrease the effectiveness of sonoporation through US-mediated endocytosis, suggesting that this phenomenon can be induced by ROS-dependent mechanisms [118].

Fewer studies addressed the role of exocytosis in US-triggered membrane repair. As mentioned above, large membrane damage is repaired through exocytosis of a patch of intracellular vehicles. When a microbubble generates pores in the cell membrane, calcium ions can enter the cytoplasm and initiate the depolymerization of actin fibers, leading to the accumulation of intracellular vehicles close to membrane disruption. Then, the vehicles can fuse with each other and create large patch vehicles, which are recruited to the cell membrane through the disrupted actin cytoskeleton. The fusion of vehicles and the cell membrane repairs the pores and restores the integrity of the cell membrane [12]. Some studies also suggested that lysosome exocytosis was caused by US-mediated membrane damage and was crucial for the enhancement of endocytosis. Fekri et al. [122] detected an increase in the cell surface level of LAMP-1—a lysosomal marker—in RPE cells after US exposure. Vacuolin-1 and desipramine—the lysosome exocytosis inhibitors—were shown to down-regulate cell surface transferrin receptor activity, implying that exocytosis compensates for sonoporation-induced endocytosis.

US-mediated pore formation facilitates the free flow of various ions through the cell membrane, including extracellular Ca^2+^. Since numerous cellular processes are controlled by Ca^2+^, some researchers investigated the role of calcium ions in membrane resealing. One of the pivotal papers that pointed out the role of calcium ions in US-induced membrane repair was published by Zhou et al. They designed an experiment where the sonoporated *Xenopus* oocytes were exposed to various calcium concentrations (0–3 mM). By monitoring the single cell resealing rate in real time, they noticed that lower Ca^2+^ concentration was associated with slower and weaker recovery, and the lowest calcium concentration enabling successful membrane repair was 0.54 mM. Moreover, membrane recovery was not possible in calcium-free solutions [111]. Deng et al. also noticed that resealing of the cell membrane was faster in the presence of extracellular Ca^2+^ [20]. Calcium ions are also believed to affect Ca-dependent proteins and cytoskeleton, possibly leading to the depolymerization of filaments. This phenomenon would be crucial for sonoporation-mediated exocytosis to enable vesicle fusion with the cell membrane [33]. Furthermore, Ca^2+^ can stimulate the lysosomes to fuse with each other [123] or the plasma membrane [124], providing a successful repair of the pores generated by US. Calcium ions can also influence cholesterol-rich cell membrane compartments and trigger their spontaneous vesiculation, leading to the formation of endocytic vesicles.

### 4.4. Lipid Peroxidation

Many researchers have reported the production of reactive oxygen species during sonoporation—this phenomenon will be discussed in Section 5.2. Some of them claimed that the generated free radicals can penetrate the lipid bilayer and induce lipid peroxidation. However, it has not yet been established whether sonoporation induces peroxidation through enzymatic or nonenzymatic reactions. This issue remains crucial on account on the fact that the final fate of the cell may differ depending on the type of process. For example, the production of oxidized lipids can affect the structure of the lipid bilayer and therefore alter the membrane properties such as permeability [125,126]. Jia et al. examined cell membrane damage of normal breast cancer cells (MCF-7) and resisted to doxorubicin (MCF-7/ADR). As assumed, the level of lipid peroxidation was remarkably increased after sonoporation; however, its intensity varied significantly between the examined cell lines. Whereas US exposure at 0.75 W/cm^2^ provided the highest level of methane dicarboxylic aldehyde (MDA) in doxorubicin resisted cells—19.170 ± 0.004 nmol/mL, the concentration of MDA in cells sensitive to doxorubicin was much lower—8.337 ± 0.003 nmol/mL. These results suggested that the cell membrane damage was much more severe in MCF-7/ADR cells. The authors claimed that the decrease in membrane fluidity of MCF-7/ADR cells would explain the increase in cell membrane damage [87]. It is worth mentioning that this is not the first observation of altered membrane fluidity of resisted cancer cells. Previous studies have reported that cell membranes of drug-resistant malignant cells are overloaded with many proteins such as P-glycoprotein, which enable the effective elimination of cytostatics. However, this phenomenon has significant biophysical consequences such as decreased membrane fluidity. Because US-based sonoporation is a mechanical process that strongly relies on biophysical features, it is highly likely that some cellular bioeffects will differ between drug-sensitive and resistant cancer cells. Going further, it seems that the occurrence of multidrug resistance can determine the intensity of lipid peroxidation. Furthermore, membrane fluidity can also be altered by temperature and this may again alter cell susceptibility to sonoporation and lipid peroxidation [107,127]. Leung et al. investigated lipid peroxidation in sonoporated Jurkat cells. The authors observed reduced levels of cholesterol, arachidonic, eicosapentaenoic, and docosahexaenoic acids, 24 h after US treatment. Furthermore, only the levels of enzyme-independent oxidized lipids (F2-isoprostanes, F3-isoprostanes, 7-ketocholesterol) were increased after sonoporation, up to 60% compared to control cells, whereas the levels of enzyme-dependent oxidized products remained stable. As far as cell morphology can be altered by cellular stress, the authors also observed membrane shrinkage and accumulation of intracellular lipids, which proved the essential disruption of lipid metabolism [128]. In addition, the cell membrane cytoskeleton is made up of crucial PUFA acids conjugated to phospholipids [125], which were reduced in sonoporated Jurkat cells. This could imply serious consequences such as altered cell surface structure (which was proven in other studies by SEM images) or even modified cell-to-cell communication. Some oxidized lipids play an important role in regulation of cell division [129] or increase membrane fluidity [130], whereas cholesterol oxidation products are highly toxic [131] and can induce cell death by overproduction of superoxide anions [132] as well as induction of nuclei fragmentation [128]. Lipid peroxidation has also been reported in ultrasonicated homogenates of Ehrlich ascitic tumor cells [130] and ghost membranes of erythrocytes in the presence of hematoporphyrin [133].

### 4.5. Membrane Protein

Although the mechanisms responsible for bubble- and non-bubble-based sonoporation significantly differ from each other, both lead to membrane rupture. As a result, US-mediated exocytosis and endocytosis can alter the biological characteristics of the cell membrane. Brayman et al. [134] reported the removal of CD19 receptors from the plasma membrane after US exposure. Although the authors did not link this observation specifically with endocytosis, a similar phenomenon was described in bacteria that removed the pores created by the bacterial protein streptolysin O by endocytosis [135]. Since multidrug resistance phenomena (MDR) are facilitated in part by membrane proteins responsible for effective drug efflux, some scientists went further and explored the effect of US exposure on MDR. Microbubble-based sonoporation has been shown to reduce the expression of Pgp in the blood-brain barrier in rats. Aryal et al. [136] observed a suppression of Pgp that lasted more than 72 h. This would increase drug penetration and accumulation in the central nervous system. The reduction in Pgp activity was also revealed in sonoporated breast cancer cells. A significant increase in drug accumulation was observed even 15 min after US treatment. Furthermore, Western Blot analyses confirmed 44.4% down-regulation of Pgp in doxorubicin resistant MCF-7/ADR cells. The authors proposed two mechanisms that could explain this observation. The decreased expression of Pgp might be caused by DNA damage during sonoporation, which suppresses transcription and translation. On the other hand, the US “shaving” effect may be also responsible for removal of extracellular domains from glycoproteins and, thus, alteration of protein activity [137]. Similar findings were reported by Bjånes et al. [138] who investigated the gemcitabine efficacy in sonoporated pancreatic cancer cells. Alternatively, drug resistance can be overcome by targeting MDR-related genes. Paproski et al. used microbubble-based sonoporation to transfect HEK293 cells and increase gemcitabine sensitivity by introducing the membrane transporter—the human concentrative nucleoside transporter 3 (hCNT3) gene. US-mediated transfection increased the expression of the hCNT3 gene by more than 2000-fold and provided a 3400-fold increase in intracellular gemcitabine uptake [139,140].

### 4.6. Cell Morphology

Cell size was shown to decrease after US treatment [141,142] and a flatter and smoother cell surface was observed [143]. SEM and confocal microscopy of sonoporated prostate cancer cells DU-145 revealed the presence of wounded cells with spherically protruding ‘balloons’ and ‘blister’ blebs. The balloon blebs contained small lipid bubbles produced by the endoplasmic reticulum, whereas the blister blebs derived from lipids fused with the cell membrane and contained clear fluid. Schlicher and co-workers [116] also observed various types of membrane blebbing, nuclear ejection, perikarya formation, or even cell lysis. Zeghimi et al. used TEM and SEM microscopy to observe the evolution of plasma membrane disruption of U-87 MG cells. They detected a large amount of uncoated pits and membrane ruptures that were almost completely resealed within 60 min [144]. However, some of the morphological alterations, such as pit-like structures and membrane roughness, did not recover until 24 after US treatment with opsonin microbubbles [34,36]. Other morphological alterations observed after sonoporation were cell membrane blebs. Qin et al. detected blebs 90 s after exposure, which later expanded and created new blebs. These formations were observed in reversibly and irreversibly sonoporated cells [16]. Moreover, Leow and co-workers [112] also mentioned the presence of secondary blebs located far from the sonoporated areas. Blebs are believed to be created to restore pressure homeostasis as a cytoprotective response to an increase in hydrostatic pressure caused by the US disruption of the actin cytoskeleton [112,145]. Honda et al. also discovered apoptosis cells 6 h after sonication. Characteristic shrinkage of the nucleus and cell body, chromatin condensation, and nucleus fragmentation were observed, but swelling of the cytoplasm and enlarged endoplasmic reticulum was present only in some dying cells. Moreover, vacuolar structures were detected and described as autophagic vacuoles and secondary lysosomes [146]. Tachibana et al. [147] mentioned the reduced number of microvilli and membranous laminar ruffles in HL-60 cells after US treatment. In the next few years, confocal images revealed a regional increase in lipid content in wounded areas, which gradually propagated outwards, and lipid striation patterns on the cell membrane of HL-60 cells subjected to US. The authors proved that the emergent lipids were derived from the intracellular environment [148]. Furthermore, sonoporated MCF-7 cells also appeared to have no microvilli after sonoporation, which resulted in their smooth cell surface, and many cells were round and shrunken. These membrane disruptions were much more intense in doxorubicin resistant MCF-7/ADR cells [87]. Swollen mitochondria and cytoplasmic vacuoles were reported in ovarian cancer cells treated with US [149]. Additionally, many sonoporated cells contained caveolar or clathrin-mediated endocytic vesicles, as previously described. Although US-mediated sonoporation is primarily associated with pore formation in the cell membrane, this phenomenon has far-reaching consequences for cell behaviour and survival. These aspects of US-triggered bioeffects will be discussed below.

## 5. Intracellular Phenomena Triggered by US

### 5.1. Cytoskeleton

Cytoskeleton fibres are responsible for maintaining cell architecture and interaction with other cells and with the extracellular matrix. Numerous studies have reported cytoskeleton alteration after sonoporation. Chen and co-workers described the immediate rupture of F-actin filaments at perforated areas of the cell membrane. Therefore, they proved that sonoporation is a phenomenon that does not act only at the cell membrane level. As mentioned above, cytoskeleton disruption is a cytoprotective process required for the translocation of intracellular vehicles necessary for successful membrane resealing. The authors also observed a long-term cytoskeleton response to US treatment, such as further disassembly of F-actin fibers in globular form (G-actin), which led to a temporary increase in the G:F actin ratio after sonoporation [145]. Although this effect could be the intentional fluidization or softening of cells, this behaviour will still trigger serious implications, such as altered cell mobility and locomotion or induction of pro-apoptotic signals [150,151]. Juffermans et al. observed the appearance of actin stress fibers in the centre of sonoporated cells compared to control cells with peripherally localized F-actin. However, they stayed only for a few minutes and disappeared in 30 min [152]. Further research confirmed that US-induced shear stress is capable of inducing cytoskeleton rearrangement that causes nuclear contraction [103,153,154]. Zeghimi et al. carried out very interesting experiments with sonoporated U-87 MG cells treated with inhibitors of actin filaments and microtubule polymerization using SEM imaging. They reported a decrease in the number of permeant structures in the presence of inhibitors [155]. Furthermore, a reduced distance between the microbubble and the cell positively correlated with the severity of cytoskeleton disassembly in HeLa cells [103]. Also, acoustic waves have been shown to open tight junctions and cell-cell contacts [156,157]. As mentioned previously, mechanical stimuli perceived by the cytoskeleton can be transduced by mechanosensors and integrins into appropriate signalling pathways and trigger a particular cell response. However, it remains unknown whether and how these stimuli are correlated with sonoporation. Cytoskeleton disruptions also affect cell locomotion. For example, sonoporation inhibited adhesion and migration of smooth muscle cells [158], whereas adhesion was found to be increased in US-treated neutrophils [159]. A similar decrease in cell migration was observed in DU-145 prostate cancer cells and hepatocellular carcinoma cell lines after sonoporation [160,161].

### 5.2. Oxidative Stress

The kinetic energy delivered by US can initiate inertial cavitation, which breaks down water molecules into hydrogen atoms and highly reactive hydroxyl radicals. This process is called water pyrolysis or sonolysis [162]. Then, these primary reactive oxygen species (ROS) can react with each other or with other molecules, creating secondary free radicals. For example, when hydrogen atoms recombine with oxygen molecules within the bubbles, a hydrogen radical is generated, which splits up into superoxide radical anion [72]. Another theory claims that superoxide ions can be produced by oscillating microbubbles forming vortex-like microstreaming and shear stress [152]. Finally, two superoxide anions rapidly fuse and form H_2_O_2_ [163,164]. Early studies showed that US can induce intracellular ROS production [163]. Moreover, recent investigations revealed that exposure of gas-filled microbubbles to acoustic waves evoked extremely high pressures and temperatures capable of generating extracellular free radicals [17,72,165]. Although balanced ROS activity is necessary for cell functioning, excessive free-radical production damages cellular organelles, damages nucleic acids, causes protein misfolding, promotes aging, and cell death. This balance can be severely disturbed in the presence of microbubbles when extracellular ROS can cause physical damage to the cell and cause oxidative stress [165]. Furthermore, increasing cavitation energies were correlated with increased shear stress and ROS production, enabling intensification of sonoporation [12,54]. This phenomenon was accompanied by a decrease in the concentration of intracellular scavengers such as glutathione [152]. Escoffre et al. [66] showed that ROS production appeared to play a supporting role in membrane permeabilization, and cavitation was the predominant process that allowed effective transfection with US. Moreover, some scientists explored the effect of catalase on oxidative stress during sonoporation. Further tests confirmed that when catalase was added, reduced US-mediated intracellular production of H_2_O_2_ was observed, but this phenomenon was accompanied by an immediate blockage of Ca^2+^ influx after exposure. These findings suggest that hydrogen peroxide plays an important role in native permeabilization of the plasma membrane and the formation of transient nanopores [119,166]. Going further, calcium ions can modulate the transmembrane current by activating BK_Ca_ stretch channels, highlighting the link between oxidative stress and cell membrane potential. On the other hand, an increased intracellular concentration of Ca^2+^ ions is known to induce the production of free radicals by mitochondria. Thus, mitochondria seem to play a role in the transduction of US-triggered oxidative stress [146,152]. Sonochemical reactions were also capable of lysis of human promyelocytic leukemia HL-60 cells [167] or changing the physicochemical characteristics of culture medium, which after sonication lost its ability to support cell development [168]. Experiments with free radical scavengers have shown that the intensity of endocytosis is also higher when ROS are produced [118,164]. Moreover, antioxidants reduced doxorubicin uptake in sonoporated cells [169]. Although US-induced oxidative stress remains an intriguing research topic, designing and carrying out relevant experiments is still a huge problem. Intracellular ROS generation was assessed mainly by fluorospectrophotometry, microscope observations, or flow cytometry at appropriate time points. The short existence of free radicals and the nature of US investigations make this issue much more complex. Research into solving this problem is still under way.

### 5.3. Endoplasmic Reticulum

**Figure 4:**
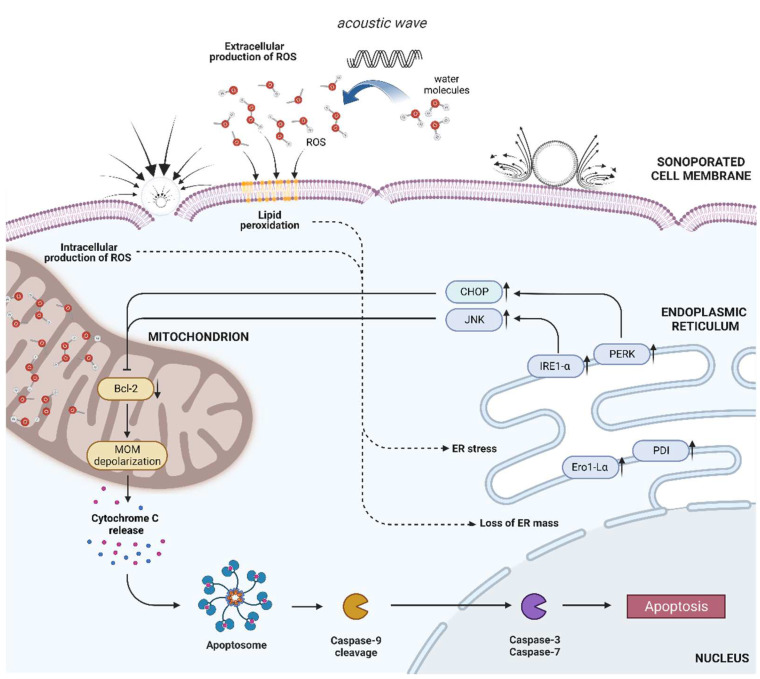
Sonoporation-triggered ER stress and apoptosis induction; up arrow—upregulation, down arrow—downregulation. Created with BioRender.com.

The endoplasmic reticulum (ER) is a complex membrane system that separates particular areas within eucaryotic cells. As previously reported, ER is involved in exo- and endocytosis and, thus, membrane repair [170]. Moreover, ER is responsible for protein folding, lipid metabolism as well as calcium signalling. Based on these findings, Zhong and co-workers hypothesized that ER could participate in the detection and mediation of US-triggered cellular stress. First, they noticed a loss of total ER mass measured by the decrease in ER-Tracker Green fluorescence intensity after sonoporation. Western Blots revealed an increase in the expression of protein folding enzymes: Ero1-Lα (ER oxidoreductin-1 like protein, α paralog) up to 202%, and PDI (protein disulfide isomerase) up to 76%. Finally, their team confirmed activation of ER stress-sensor proteins: PERK (protein kinase RNA-like ER kinase) and IRE1-α (inositol-requiring protein-1, α homolog). In that way, they concluded that loss of ER mass accompanied by activation of stress sensors seriously disrupted the ER in US-exposed cells. This phenomenon usually leads to the accumulation of misfolded proteins, which was also confirmed in this study. Furthermore, when ER stress is extended and cells cannot adapt to this, the stress signals are transduced and initiate ER-induced apoptosis. As expected, the authors recognized the activation of pro-apoptotic regulators in the ER: CHOP and JNK, which are known to inhibit antiapoptotic Bcl-2 protein and induce mitochondrial membrane depolarization. This triggered an intrinsic apoptotic pathway in HL-60 cells that was confirmed by caspase-9 cleavage. Zhong et al. revealed that sonoporation is not only capable of ER stress induction but also that these signals can be transduced to mitochondria and guide US-treated cells toward apoptosis. Unfortunately, their research is the only existing paper that thoroughly addressed the functioning of the ER of sonoporated cells [171]. Only some other studies mentioned ER disturbances in US-treated cells [146,148,172]. The functioning of the endoplasmic reticulum in cells exposed to US remains a very exciting issue, since ER seems to play a crucial role in cell response to sonoporation, especially considering its link with mitochondria in cell death induction. The Golgi apparatus was also not investigated in sonoporated studies; however, it could cooperate with the ER in lipid secretion and membrane resealing. Research into solving this problem is still under way. The graphical summary of ER-induced stress and cell death is presented in Figure 4.

### 5.4. Mitochondria

Mitochondria, commonly called the powerhouse of the cells, were noted as the intracellular sensors and transducers of US-induced stress by increasing the production of reactive oxygen species [146,152] or altering calcium signalling [33]. TEM imaging confirmed apical membrane widening, as well as mitochondrial cristae disruptions in sonoporated frog-muscle fibers and epidermis of tadpoles [173,174]. Additionally, mitochondrial swelling was observed in sonoporated ovarian cancer cells [149]. Zhong et al. [171] also reported the depolarization of the mitochondrial outer membrane (MOM) using JC-1 staining: the MOM potential of sonoporated cells gradually decreased from its initial value to a depolarized state. This phenomenon is mainly caused by extended cytoplasmic stress that promotes cytochrome c release and the induction of mitochondria-dependent apoptosis [175], as revealed in sonoporated cells [19,176,177,178]. Furthermore, sonoporation was also accompanied by mitochondrial superoxide production and oxidative stress induction [146].

### 5.5. Extracellular Vesicles

Many cells have been shown to excrete small extracellular vesicles (EVs). EVs are natural phospholipid bilayer structures that are classified into different groups based on their size and biogenesis mechanism: exosomes (30–120 nm), microvesicles (50–1000 nm) and apoptotic bodies (50–2000 nm) [179]. EVs were first viewed as cellular debris, but are now thought of as vesicles with diverse biological functions, including removal of waste products from cells, cell membrane repair, emission of signalling and regulatory molecules, cell-cell communication, regulation of the immune system, and antigen presentation [180]. Yuana and co-workers tried to investigate whether US exposure triggered the release of EVs from FaDu head and neck cancer cells. They found an increased level of CD9 exposing-EVs at 2 and 4 h and CD63 positive-EVs at 2 h following the treatment. After 24 h, EVs’ secretion reached their initial intensity. However, the isolated EVs displayed a nonhomogeneous size distribution profile (30–1200 nm) and the presence of calnexin, which is known to be absent in exosomes. Thus, sonoporation induced exocytosis and release of heterogeneous EVs [181]. Interestingly, exposure to US increases intracellular calcium concentration, which can trigger exocytosis and thus EVs’ secretion during membrane resealing.

### 5.6. Calcium Signalling

Calcium signalling seems to play a crucial role in the functioning of sonoporated cells. In addition, they can modulate the transmembrane potential by activating particular ion channels and regulating oxidative stress. Ca^2+^ are essential for successful membrane recovery through US-induced exocytosis. Calcium-enhanced US-transfection efficiency was also reported in NIH3T3 cells by activation of plasmid endocytosis [182]. Intracellular increase in calcium concentration can be caused by free Ca^2+^ influx through generated membrane perforations or Ca^2+^ release from intracellular storages such as the endoplasmic reticulum, mitochondria, or buffering proteins—this mechanism is triggered by secondary messengers of PLC-IP_3_-IP_3_R axis induced by mechanical stimuli [183,184]. To confirm this, Takahashi and co-workers inhibited PLC and IP_3_R in sonoporated cells and observed a reduced increase in intracellular calcium. Similar observations were noted when extracellular calcium ions were depleted. Therefore, the increase in intracellular Ca^2+^ in cells treated with US was caused by the influx of extracellular Ca^2+^ as well as intracellular Ca^2+^ release. Since Ca^2+^ is responsible for cytoskeletal filaments’ rearrangement [31], their team also investigated the role of cytoskeleton in increasing intracellular calcium concentration. Further tests showed that only actin fibres were involved in US mechanosensing and an increase in intracellular Ca^2+^ was required for induction of US mechanotransduction [185]. Real-time observations of sonoporated cells revealed that intracellular calcium fluctuations appeared up to 45 s following exposure and lasted for up to 3 min [152,166,186]. On the other hand, Honda et al. [146] observed a rise in intracellular calcium concentration for 4 h that reached the initial level at 6 h after US treatment. Some cells also experienced delayed ultrashort frequency-modulated Ca^2+^ oscillations that were thought to be cellular transmitters responsible for modulation of calcium-sensitive targets and cell adaptation to US [33]. Calcium ions also regulate the nuclear envelope and nuclear pore complexes (NPCs). The study of Vaškovicová et al. [187] highlighted that US affected NPCs of HL-60 cells which had wider diameters following the treatment. That would also significantly increase the permeability of nuclear envelopes for medicines and genes. Taking into account the above findings, this observation could be explained by calcium fluctuations induced by US. Finally, calcium-mediated sonoporation was found to induce rapid cell death of CHO cells within 20 min after US-exposure. Until now, Maciulevičius and co-workers remain the only team to have investigated the effect of calcium sonoporation on cell viability. In contrast to other studies [20,111], they did not observe a protective effect of Ca^2+^ at low Ca^2+^ concentration (<3 mM). Furthermore, only healthy cells were used in their research, so it is still unknown how such therapy would affect malignant cells.

### 5.7. Signalling Pathways

A signalling pathway is a series of biochemical reactions in which a particular group of molecules work together to control cell functions such as cell division or apoptosis. When a substance, such as a hormone or growth factor, attaches to a specific protein receptor presented on the surface or within the cell, the signal is transduced using secondary messengers and finally activates molecular targets that trigger intended cellular responses. Signalling pathways steer the cellular biochemical machinery and are responsible for homeostasis maintenance, adaptation to stressful conditions, and cell recovery or cell death induction if necessary.

Only a few studies have addressed the regulation of the signalling pathway in sonoporated cells. Haugse and co-workers investigated the effect of BBS on the activation of important intracellular signalling pathways in pancreatic cancer cells (MIA PaCa-2), fibroblasts (FB) and human-umbilical-vein endothelial cells (HUVECs). In all three cell types, sonoporation led to phosphorylation of MAP-kinases (MAPK): p38 T180/Y182 and ERK1/2 T202/Y204; however, the intensity and time of activation differed among particular cell lines. The highest MAPK activation was noticed in fibroblast directly after US exposure, but in MIA PaCa-2 and HUVECs this process was weaker or delayed. Moreover, mTOR pathway activation with phosphorylation of ribosomal protein S6 was reported in MIA PaCa-2 and FB 2h following sonoporation. US also induced the dephosphorylation of 4E-BP1 T36/45. Since ERK 1/2 activation has been shown to play an important role in injured recovery, their team investigated whether EKR 1/2 inhibition would affect cellular response to US. As suspected, this increased the percentage of apoptotic cells in HUVECs and FB cell lines, but not in MIA PaCa-2 cancer cells. The authors claimed that MAPK activation and inhibition of 4E-BP1 could be the signalling response of sonoporated cells to membrane repair [188], which was observed in electroporated cells [189,190]. Furthermore, dephosphorylation of 4E-BP1 was shown to inhibit cap-dependent protein translation [191]. Haugse et al. also investigated the activation of signalling pathways in US-treated leukemic MOLM-13 cells and peripheral blood mononuclear cells (PBMC). In contrast to their previous study, this time phosphorylation of p38 T180/Y182, ERK1/2 T202/Y204, CREB S133/ATF-1, Akt S473 and STAT3 S727 was present only in MOLM-13 cells, but not in PBMC. In agreement with their findings, other investigations also noted that US could activate p38, ERK, and Akt [192,193,194,195]. Such proteins have been reported to regulate cell cycle and proliferation [196], cell stress [197], cell survival [198] as well as many other processes.

Sato et al. [194] described US-induced activation of integrin receptors—focal adhesion kinase (FAK), which was followed by phosphorylation of up-regulation of ERK 1/2, p38 and JNK in rabbit-knee synovial-membrane cells. Moreover, Whitney et al. [192] confirmed the phosphorylation of Src by activated FAK in sonoporated cells. Zhou and co-workers revealed that US induced F-actin polymerization, paxillin recruitment to focal adhesions by activation of Rho kinase [195], which is involved in cytoskeleton reorganization [199]. Focal adhesions (FAs) are multiprotein structures that form physical links between integrin cytoplasmic domains and cytoskeleton fibres. FAs modulate signalling pathways associated with integrins, such as the MAPK and Rho pathways. Taken together, these findings suggest that FA and integrin-mediated pathways are responsible for mechanotransduction of biophysical stimuli triggered by sonoporation and initiate the cascade of cellular responses leading, for instance, to membrane repair, cell cycle alteration, cytoskeleton rearrangement, or even cell death.

### 5.8. DNA Damage

Previous studies have shown that DNA damage is responsible for many human diseases, including cancer. DNA disruptions occur naturally and daily in our cells, which are constantly exposed to extrinsic or intrinsic factors capable of introducing defects in one DNA strand (single strand breaks—SSB) or both (double strand breaks—DSB). DNA repair is provided by genetically programmed complexes that are responsible for correcting the erratically installed nitrogenous bases (base excision repair—BER) or defective portions (nucleotide excision repair—NER). Unfortunately, in some cases, cells cannot restore the primary genetic balance that initiates programmed death in healthy cells [22]. The ability of US to affect the DNA structure was proved many years ago. Until now, chemical and mechanical reactions have been believed to participate in sonoporation-mediated changes in genomic DNA [200,201]. Moreover, the mechanical stimuli provided by US exposure can be transduced and trigger appropriate effector mechanisms, which consequently modulate gene expression [202,203].

SSB are the most common lesions found in DNA, which can be caused directly by ROS or indirectly by enzymatic activity during BER. Early evidence of SSB were revealed in 1977 by McKee and co-workers who noticed strand breaks in the DNA solutions subjected to UD. The authors claimed that the production of free radicals during the sonolysis of water molecules was responsible for this phenomenon [204]. In 1986, Pinamonti et al. [205] reported SSB in sonoporated human leukocytes. Later experiments have shown that intracellular ROS production is critical for provoking SSB whereas extracellular free radicals did not alter SSB intensity due to the short existence and inaccessibility of the nucleus for ROS outside the cell [206,207,208].

**Figure 5:**
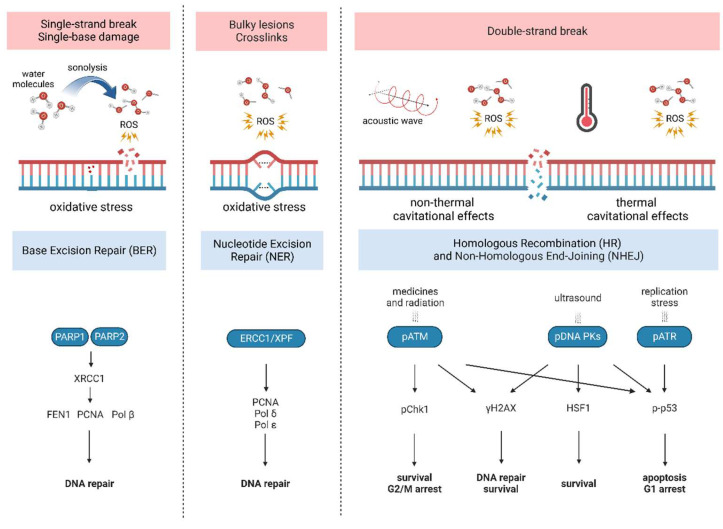
An overview of US-induced DNA damage mechanisms and cellular response. Created with BioRender.com.

Double-strand breaks are, though, the most hazardous DNA disturbances, as the lack of original template copy results in a random repair of the lesion. First records of US-triggered DSB were reported by Kondo et al. in 1985 [209]. Later, they were confirmed in various leukemia cell lines using a neutral comet assay [22]. Interestingly, the molecular signalling of sonoporation-evoked DSB DNA damage varies from pathways triggered by ionizing radiation (IR). When DSB are detected, the histone H2AX is phosphorylated (γH2AX) which is a platform for DNA damage response proteins responsible for DNA repair. H2AX phosphorylation in S139 is the result of the activation of PIKK family proteins such as ataxia-telangiectasia-mutated (ATM), DNA-dependent protein kinase (DNA-PK) as well as ataxia telangiectasia and Rad3-related protein (ATR). Thus, γH2AX is considered a cellular sensor of DSB and its presence was confirmed in sonoporated cells [22]. ATM is generally activated by medicines or IR, and ATM by replication stress [210,211,212]. ATM interacts with the MRE11/RAD50/NBS1 (MRN) complex that recognizes DSB and provides homologous recombination (HR). DNA-PK is recruited with the Ku70/Ku80 heterodimer that secures DNA breaks from damage and contributes to non-homologous end-joining (NHEJ) repair. Activated ATM, DNA-PK and NBS1 proteins were confirmed in sonoporated cells [213]. However, recent studies highlighted that sonoporation preferably activated DNA-PK rather than ATM and DNA-PK phosphorylated in S2056 (pS2056-DNA-PK) was present in the peripheral areas of the nuclei and colocalized with γH2AX. Furthermore, DNA-PK phosphorylated in S2609 was detected independently from γH2AX. Therefore, pS2056-DNA-PK is believed to enable NHEJ repair of US-triggered DBS and transduce DNA-damaged signals by activating H2AX. This process is presented in Figure 5.

Since acoustic waves carry kinetic energy through cells, US can also induce heat damage to DNA by ROS production and denaturation of proteins associated with DNA. However, heat stress-activated ATM in preference to ATR and DNA-PK. The work of Abdollahi et al. sheds new light on this issue. Their team compared the efficacy of sonoporation in lymphoblasts with and without the *p53* gene. In that way, they observed that US effectively killed p53+ as well as p53 cells; however, p53 cells were more resistant to induction of apoptosis [178]. Furthermore, Furusawa et al. demonstrated that p53 phosphorylation in Ser15 is related to ATM activity and ATM inhibition resulted in suppression of apoptosis only in p53+ cells. On the other hand, DNA-PK is responsible for Akt phosphorylation and DNA-PK inhibitor-suppressed sonoporation-mediated death in p53+ and p53-. Based on these findings, the authors claimed that DNA-PK might be an interesting target for US-targeted anticancer therapy [214]. In agreement with all the presented results, DNA replication was found to be significantly lengthened even up to 250% in US-exposed cells proving serious DNA damage [141]. Hassan and co-workers also mentioned that sonoporation could promote neotic division, especially in p53-mutated cells, as a way to extend the cellular life span and thrive resistance to harmful factors [215].

### 5.9. Cell Cycle

**Figure 6:**
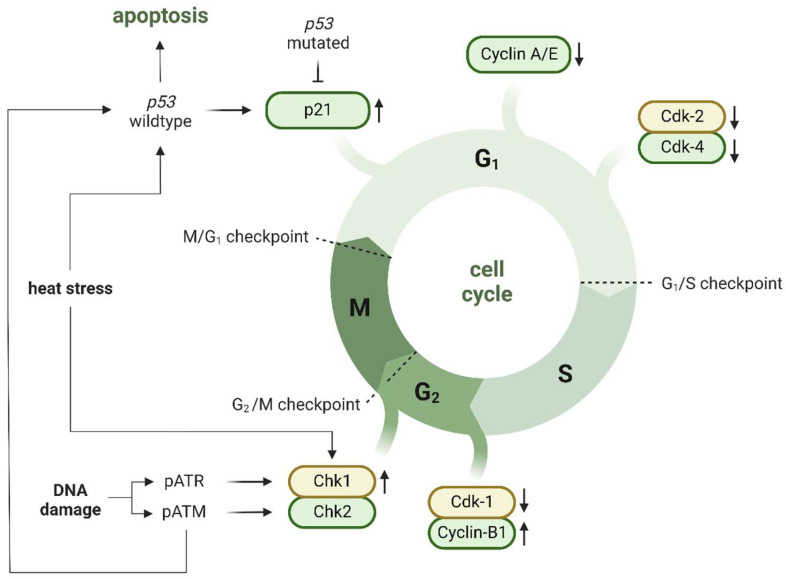
Cell cycle regulation in sonoporated cells; up arrow—upregulation, down arrow—downregulation. Created with BioRender.com.

A cell with damaged DNA cannot develop further until DNA repair is finished. Therefore, it is not a surprise that the cell cycle is altered in US-treated cells with disturbed genetic material. Cell cycle promotion is regulated by G1, S and G2/M checkpoints: the G1 checkpoint is primarily controlled by cyclin-dependent kinase inhibitor 1A (CDKN1A also known as p21)—a downstream transcript of p53, the G2/M checkpoint by checkpoint kinase 1 and 2 (Chk1 and Chk2) which are regulated by ATR and ATM (the activity of the S checkpoint is not so obvious and will not be discussed in this paper) [216,217,218]. Since numerous types of cancer cells display p53 mutations or depletion, cells with damaged DNA depend mainly on G2/M checkpoint to provide DNA repair. Zhong et al. [19] revealed that sonoporated HL-60 cells showed cell cycle arrest in G2/M phase caused by down-regulation of Cdk1 (the lowest at 4 h) accompanied by up-regulation of cyclin-B1 (the highest at 12 h). One year later, Furusawa et al. [219] reported that Chk1 provided G2/M cell cycle arrest in US-exposed Jurkat cells. Interestingly, ATR inhibitors attenuated Chk1 activity, demonstrating that Chk1 phosphorylation depended on ATR activity [220]. Moreover, Chk1 inhibition successfully minimized the cell population in G2/M phase following sonoporation, showing that Chk1 is crucial for cell survival and G2/M cell cycle arrest after US-triggered DNA damage. The role of Chk2 in post-US cell cycle regulation remains unclear. Since ATR and ATM preferentially activate Chk1 and Chk2, respectively [221] and because ATM and Chk2 can activate p53, the role of the ATM-Chk2 axis in p53-induced apoptosis and cell cycle arrest should be addressed in future research [214]. Heat stress can induce cell cycle arrest by p21 upregulation in G1 phase and by the ATK-Chk1-CDC25A pathway in G2/M phase; however, these phenomena have not yet been proven in sonoporated cells [214]. Zhong et al. also reported a suppressed level of Cdk-2, cyclin-A and cyclin-E 8 and 12 h following US treatment, which triggered G1/S phase transition and S phase progression. That observation was highlighted in the decrease of G1 and S populations of US-exposed HL-60 cells. Additionally, they noted the suppressed level of Cdk-4 which normally binds to cyclins D1/D2, but their activity kept stable in sonoporated HL-60 cells. The author concluded that the altered Cdk4 initiated by sonoporation partially contributed to a delay in the progression of the G1 phase [19]. Although the observed cell cycle alterations appear quickly and peak at the highest intensity around 12 h after sonoporation, many papers have shown that cells were able to restore their initial balance app. 24 h after treatment [22]. Cell cycle regulation induced by sonoporation is shown in Figure 6.

### 5.10. Cell Death

Acoustic waves have been reported to alter cell functioning at various levels. Although cell reaction to US strongly depends on the primary energy of sound wave, if the cell fails to overcome US-induced dysfunctions such as membrane permeabilization, ineffective membrane resealing, cytoskeleton disintegration, oxidative stress, or DNA damage, the programmed cell death pathway is initiated. Numerous studies described US-induced apoptosis of human cells and presenting this issue is beyond the scope of this study. Thus, the general findings regarding cell death triggered by sonoporation will be presented.

Several studies mentioned the presence of various cell subpopulations after US exposure [16,26,108,156]. Since US can cause reversible or irreversible sonoporation, the mechanical destruction of particular cells is so intense directly after treatment that the cells lose their integrity and undergo necrosis. The fate of the remaining cells still depends on the severity of the damage. Flow-cytometry experiments of calcein uptake revealed subpopulations with low and high accumulation of the dye. This phenomenon is a true consequence of the nature of sonoporation experiments. Although scientists are trying to control the experimental conditions as much as possible, the physical features of acoustic waves make it impossible to prevent the existence of local disturbances in the spatial distribution of acoustic kinetic energy. Thus, the occurrence of cell subpopulations after sonoporation is a natural phenomenon that determines the level of absorbed energy and the final biological outcome. Consistent with this effect, some scientists noticed that only a part of the sonoporated cells undergo apoptosis [19,222]. Sonoporated cells can try to avoid apoptosis by inducing autophagy, as mentioned by Wang et al. [223]. The molecular mechanisms of US-induced apoptosis were a subject of many studies [145,165,171,224]. Sonoporation has been shown to provoke the intrinsic apoptotic pathway by cytochrome c release from mitochondria [177], down-regulation of the pro-apoptotic Bcl-2 protein [178] and up-regulation of pro-apoptotic Bax with maximum activity 4 h after US treatment [19]. It is worth noting that apoptosis induction was intensified when microbubbles were used [225,226]. The abovementioned alterations in integrin-mediated pathways and induction of ER stress undoubtedly play an important role in US-triggered apoptosis. However, the appearance of apoptosis was not correlated with ROS induction, suggesting that mechanical destruction by inertial cavitation activity is the predominant effect triggering cell death [176]. On the other hand, US-mediated cell lysis was provided by sonochemical reactions induced by US [167]. Until now, sonoporation-activated extrinsic apoptosis has not been reported.

## 6. Conclusions

Numerous studies provided us with strong evidence that sonoporation may facilitate cancer treatment, but we are the first to evaluate the biological vastness of sonoporation-provoked effects. This review has clearly highlighted that ultrasound (US)-triggered bioeffects are not limited to the cell membrane, but they also alter cell functioning at diverse levels. Moreover, we presented a detailed biophysical insight into the process of sonoporation. Scientists should keep in mind that US exposure not only provides successful drug or gene delivery, but also induces the cascade of biochemical reactions leading to cell recovery and adaptation, or cell death. Although much research has been performed to understand how US can induce membrane permeabilization, further study is needed to adapt what has been learnt in controlled laboratory settings to clinical application. Significant benefits of US-mediated therapy with microbubbles would include its non-invasiveness, low toxicity, targeted and local application, easy adaptability, cost effectiveness, and the possibility of establishing imaging-guided therapy. Until now, there has been only one clinical study on the application of sonoporation to improve chemotherapy of pancreatic ductal adenocarcinoma [227]. This shows an unfortunately low popularity and unawareness of US-targeted therapies among clinicians. Despite the encouraging outcomes so far, some of the observed phenomena are still not fully understood or explained. For now it appears extremely challenging to find the best medicine combination and dosage, drug delivery method, microbubble and US settings as well as treatment schedule, especially taking into account the fact that particular tumors require appropriate treatment plans. Our analysis showed us that one of the biggest issues concerning US studies is the lack of methodological coherence between scientists: the more research is performed, the less we know about how selected US doses triggerring particular cell responses in various types of cells. Therefore, specifying a standardized research methodology would considerably improve our understanding of sonoporation-induced bioeffects and would allow us to design strategies to control and maximize US-enhanced drug delivery and therapies. Since comprehensive sonoporation studies require a close cooperation between US physicists, drug-delivery chemists and pharmacists, biologists and physicians, US-related research is expected to develop in the upcoming years as the multidisciplinary approach is gaining increasing attention and popularity in science.
